# Triglyceride Glucose–Body Mass Index Is a Reliable Indicator of Bone Mineral Density and Risk of Osteoporotic Fracture in Middle-Aged and Elderly Nondiabetic Chinese Individuals

**DOI:** 10.3390/jcm11195694

**Published:** 2022-09-26

**Authors:** Zhangxin Wen, Yongfang Li, Lulu Xu, Chun Yue, Qinyi Wang, Rong Chen, Na Ding, Xiaoli Qu, Yangna Ou, Yanyi Yang, Zhifeng Sheng, Hong Liu

**Affiliations:** 1 Health Management Center, National Clinical Research Center for Metabolic Diseases, Department of Metabolism and Endocrinology, Hunan Provincial Key Laboratory for Metabolic Bone Diseases, The Second Xiangya Hospital of Central South University, 139 Middle Renmin Road, Changsha 410011, China; 2 Department of Metabolism and Endocrinology, The Affiliated Zhuzhou Hospital of Xiangya School of Medicine, Central South University, 116 Changjiang South Road, Zhuzhou 412007, China; 3 Health Management Center, The Second Xiangya Hospital of Central South University, 139 Middle Renmin Road, Changsha 410011, China; 4 Hospital Infection Control Center, The Second Xiangya Hospital of Central South University, 139 Middle Renmin Road, Changsha 410011, China

**Keywords:** triglyceride glucose–body mass index, bone mineral density, femoral neck geometry, osteoporosis, fracture, elderly

## Abstract

(**1**) **Background:** This study aimed to investigate the relationship of triglyceride glucose–body mass index (TyG-BMI) with bone mineral density (BMD), femoral neck geometry, and risk of fracture in middle-aged and elderly Chinese individuals. (**2**) **Methods:** A total of 832 nondiabetic individuals were selected from the prospective population-based HOPE cohort. All individuals underwent DXA for assessment of BMD at the lumbar spine, femoral neck, and total hip, as well as femoral neck geometry. The 10-year probabilities of both major osteoporotic (MOFs) and hip fractures (HFs) were calculated. (**3**) **Results:** Cortical thickness, compression strength index, cross-sectional moment of inertia, cross-sectional area, section modulus, and 25(OH)D levels were significantly lower in women (all *p* < 0.001). The presence of osteoporosis was related to age, BMI, BMD and femoral neck geometry, TyG-BMI, MOF, and HF. TyG-BMI was positively correlated with BMD. In men, TyG-BMI showed significant negative correlation with HF but not with MOF, the correlation exists only after adjusting for other variables in women. Femoral neck geometries were significantly impaired in individuals with low TyG-BMI. (**4**) **Conclusion:** TyG-BMI is positively associated with BMD and geometry, and negatively associated with risk of fracture in nondiabetic middle-aged and elderly Chinese men and women.

## 1. Introduction

Osteoporosis is a systemic skeletal disease characterized by low bone mass and microarchitectural deterioration of bone tissue, with resulting increased bone fragility and high risk of fracture [1]. It is a major contributor to the global burden of disease, and early recognition and management will benefit both individuals and society. Bone mineral density (BMD), determined by dual-energy X-ray absorptiometry (DXA), is currently widely used to assess the quality of bone. However, only 50–70% of total bone strength can be attributed to BMD. Previous research has shown that some patients with type 2 diabetes mellitus (T2DM) and obesity have high fracture risk despite having high BMD [2,3,4,5]. Independent of BMD, bone geometry contributes to fracture risk [6,7,8]. Geometric parameters of the femoral neck, such as cross-sectional area (CSA), buckling ratio (BR), and section modulus (SM) [9] also describe bone strength and are independently predictive of hip fragility fracture [10,11,12]. 

Insulin resistance (IR) does not seem to have a detrimental effect on bone mass, the most important parameter for the diagnosis of osteoporosis [13,14]. Animal research suggest that insulin exerts an anabolic effect on bone and is a critical regulator of skeletal development and structural integrity [15,16]. In the Rotterdam Study, which enrolled nearly 6000 elderly men and women, higher glucose and insulin levels were associated with higher bone mass at all skeletal sites, supporting the association between increased insulin levels and high BMD [17]. Abrahamsen and colleagues [18], however, found no association between BMD and insulin, but this may have been due to the relatively small population studied. In addition to BMD, femoral neck geometry affects bone strength. Studies have reported an inverse association between femoral neck strength and IR [19], but the study populations included individuals with and without diabetes. Shanbhogue et al. [20] reported that hyperinsulinemia directly affects bone structure, independent of obesity, in nondiabetic postmenopausal women. A few studies have examined the relationship between insulin resistance and osteoporosis in nondiabetic patients. Francisco et al. [21] conducted research in nondiabetic postmenopausal women and showed that there is a direct relationship between IR and BMD, but no association between IR and the prevalence of osteoporosis. It is possible that the relationship between IR and bone metabolism varies with sex, race, and bone mass or structure.

IR was earlier evaluated by the pancreatic suppression test, the hyperinsulinemic euglycemic clamp technique (HEGC), or the minimal model approximation of the metabolism of glucose [22,23,24]. However, these methods are invasive, complicated, expensive, and difficult to use in the clinical setting. Meanwhile, the homeostasis model assessment for insulin resistance (HOMA-IR), which is mostly used nowadays, is limited by the absence of consensus on the reference value. Recently, the triglyceride and glucose–body mass index (TyG-BMI)—which incorporates fasting blood glucose, serum triglyceride levels, and body mass index (BMI)—has been proposed as a reliable and highly sensitive and specific alternative marker of IR [25,26]. Several studies have shown that high TyG-BMI is associated with cardiovascular events [27] and incident nonalcoholic fatty liver disease in a healthy population [28], and Khamseh et al. found that TyG-BMI is a reliable discriminator of liver fibrosis [29]. However, to date, no clinical studies have examined the association between TyG-BMI and bone metabolism. This study aimed to investigate how TyG-BMI is related to BMD, femoral neck geometry, and risk of fracture in nondiabetic Chinese middle-aged and elderly individuals.

## 2. Patients and Methods

The study population was selected from among the participants of the HOPE study, an ongoing prospective study that is enrolling individuals undergoing physical examination at the Health Management Center of Xiangya Second Hospital. The HOPE study, which aims to achieve a sample size of 5000 over a period of 1 year, has already accumulated more than 1800 patients. Patients are eligible for enrollment in the HOPE study if they (1) are ≥40 years old and (2) undergo DXA for BMD measurement. The exclusion criteria are (1) history of hip joint replacement or lumbar spine surgery; (2) inability to undergo DXA for any reason; (3) history of treatment with antiosteoporosis drugs; or (4) history of malignant tumor. 

For the present study, we selected 832 healthy postmenopausal women and men aged ≥50 years from the HOPE cohort. We excluded patients with diabetes mellitus and individuals without data on fasting blood glucose or serum triglycerides. The medical records of the selected patients were searched to obtain details such as age, years since menopause, investigation results, height, and weight. BMI was calculated as the ratio of weight (in kilograms) and height (in meters) squared (kg/m^2^). This study was approved by the Ethics Committee of Xiangya Second Hospital, South China University, Changsha, China (approved number LYF2021015).

DXA scans were performed by two experienced physicians using the bone densitometer (Discovery Wi S/N87556; Hologic, Marlborough, MA, USA). The quality control (QC) program at a DXA facility includes adherence to manufacturer guidelines for system maintenance. In addition, reliability analysis was according The International Society for Clinical Densitometry guideline. The regions scanned were the left femoral neck, total hip, and lumbar spine. Seven hip geometric parameters were calculated: outer diameter, CSA, cortical thickness (CT), cross-sectional moment of inertia (CSMI), compression strength index (CSI), SM and BR (from BMD), and areal bone size data. Outer diameter refers to the outer diameter of the femoral neck at its midpoint. Endocortical diameter refers to the endocortical diameter of the femoral neck at the midpoint. CSA is an index of axial compression strength and reflects the resistance to loads directed along the bone axis. CSMI is a measure of the mass distribution relative to the geometric center; it reflects how effective a cross-section is at resisting bending and torsion—depending on the axis chosen for calculation. CT is an estimate of mean cortical thickness. BR is an index of bone geometric instability and reflects the resistance against compressive stress, which could lead to sudden sideways deflection of the structural member; higher BR values indicate greater instability and higher fracture risk [30,31,32]. CSI is a measure of the ability of the femoral neck to withstand compressive load in the axial dimension. SM, computed as CSMI divided by the distance from the bone edge to the centroid, describes femoral neck bending strength. A China-specific fracture risk assessment tool (FRAX) algorithm (which included the femoral neck BMD T-score) was used to determine the 10-year probability of major osteoporotic fractures (MOFs) and hip fractures (HFs).

Blood samples were obtained after enrollment and overnight fast of at least 8 h. Fasting blood glucose, serum triglycerides, serum total cholesterol, and serum high-density lipoprotein cholesterol were measured using an ADVIA 1650 Chemistry Analyzer (Siemens, Washington, DC, USA), and a Hitachi 7600 Automatic Analyzer (Hitachi, Tokyo, Japan). Serum levels of total 25-hydroxyvitamin D (25OHD) were measured by an automated chemiluminescence system. TyG-BMI was calculated using the formula [33] TyG-BMI = Ln [fasting glucose (mg/dL) × triglycerides (mg/dL)/2] × BMI.

### Statistical Analysis

Continuous data were assessed for normality and analyzed using the independent samples *t*-test or the Mann–Whitney *U* test, as appropriate. Pearson or Spearman correlation was used to examine associations between TyG-BMI and BMD, femoral neck geometry, and risk of fracture. Participants were stratified by sex to examine sex-specific associations. Multivariable linear regression analysis was used to explore associations between TyG-BMI and femoral neck parameters (bone density and femoral neck geometry) in the two sexes. Multivariable logistic regression analysis was used to explore associations between TyG-BMI and osteoporosis. All analyses were performed using SPSS 26 (IBM Corp., Armonk, NY, USA). Statistical significance was at *p* < 0.05.

## 3. Results

### 3.1. Clinical Characteristics of the Study Population

Characteristics of the 832 individuals (474 men, 358 women) are summarized in Table 1 and Table S1. Mean age was comparable between men and women (59.0 ± 7.95 years vs. 59.6 ± 7.72 years, *p* = 0.126). Mean TyG-BMI was significantly higher in men than in women (219.6 ± 32.5 vs. 202.5 ± 29.8, *p* < 0.001). The prevalence of osteoporosis (as diagnosed by the BMD T-score) was higher in women than in men (20.1% vs. 9.5%, *p* < 0.05). Other detailed baseline characteristics are presented in Table S1. Compared to the nonosteoporotic group, the presence of osteoporosis was related to age, BMI, BMD and femoral neck geometry, TyG-BMI and TyG index, MOF, and HF in both sexes (Table 1).

### 3.2. Association of TyG-BMI with BMD

**Figure 1:**
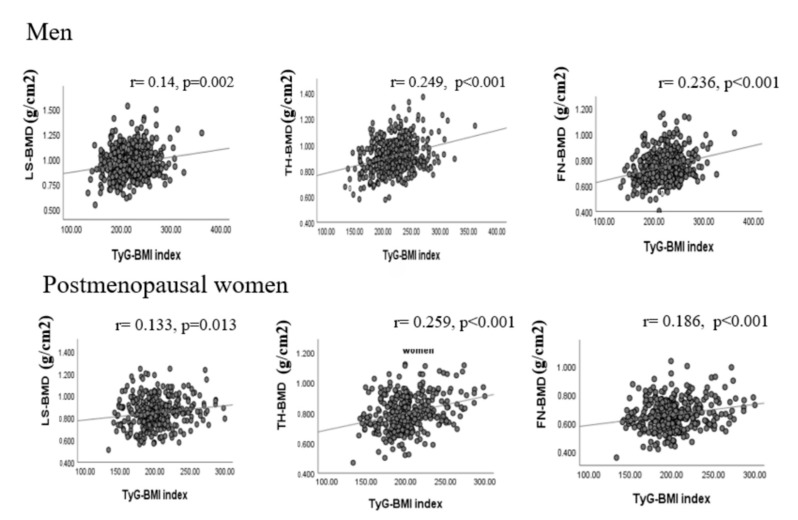
Correlations of serum TyG-BMI level with BMD. FN: femoral neck; TH: total hip; LS: lumbar spine; BMD: bone mineral density; TyG: triglyceride glucose index, TyG-BMI: combined TyG and BMI.

Figure 1 shows the correlation of TyG-BMI with BMD at different sites. In men, TyG-BMI was positively correlated with femoral neck BMD (r = 0.236, *p* < 0.001), total hip BMD (r = 0.249, *p* < 0.001), and lumbar spine BMD (r = 0.145, *p* = 0.002). Similarly, in women, TyG-BMI was positively correlated with femoral neck BMD (r = 0.186, *p* < 0.001), total hip BMD (r = 0.259, *p* < 0.001), and lumbar spine BMD (r = 0.133, *p* = 0.013). In both sexes, the association persisted even after adjusting for age, smoking, drinking, and history of previous hip fracture or parental hip fracture.

In unadjusted analysis (Table 2), TyG-BMI was positively correlated with femoral neck CSA, CSMI, SM, and CT in both men and women (all *p* < 0.001), but negatively correlated with BR and CSI. In both sexes, the associations remained statistically significant even after adjusting for age, smoking, drinking, and history of previous hip fracture or parental hip fracture (Table 3). In men, TyG-BMI was significantly correlated to HF but not to MOF; in women, TyG-BMI was not significantly correlated with either of the two factors (*p* > 0.05). However, in women, after adjusting for age, smoking, drinking, and history of previous hip fracture and parental hip fracture, TyG-BMI was significantly associated with MOF and HF; in men, the TyG-BMI remained significantly associated with HF.

### 3.3. Multiple Linear Regression Analysis of Association between TyG-BMI and BMD and Bone Geometric Parameters

Linear regression analysis showed that TyG-BMI was a significant independent predictor of BMD, SM, CSA, CSI, and BR. The standardized regression coefficients of TyG-BMI in men (Table 3) and women (Table 4), respectively, were 0.234 (*p* < 0.001) and 0.279 (*p* < 0.001) for femoral neck BMD, 0.176 (*p* < 0.001) and 0.192 (*p* < 0.001) for total hip BMD, 0.238 (*p* < 0.001) and 0.283 (*p* < 0.001) for CSA, 0.212 (*p* < 0.001) and 0.228 (*p* < 0.001) for SM, and −0.202 (*p* < 0.001) and 0.282 (*p* < 0.001) for BR.

### 3.4. Distributions of the TyG-BMI According to the Bone Health Status

**Figure 2:**
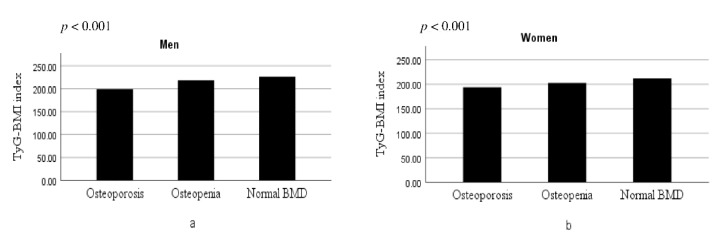
Distributions of the TyG-BMI according to the bone health status in men (**a**) and in women (**b**).

In the evaluation of the relationship between TyG-BMI and the bone health status, patients with osteoporosis had a lower TyG-BMI (*p* for trend = 0.006) compared to the others, showing a dose–response behavior (Figure 2).

### 3.5. Multivariable Logistic Regression Analyses between Possible Predictors and Osteoporosis

In the multivariable logistic regression analyses, the TyG-BMI was found to be significantly associated with osteoporosis (adjusted odds ratio (aOR) = 1.019; 95% confidence interval (CI) = 1.01–1.028) after adjusting for confounders. Age (aOR = 0.919; 95% CI = 0.892–0.947) and sex, female (aOR = 0.489; 95% CI = 0.266–0.889) were also related to osteoporosis (Table 5).

## 4. Discussion

This is the first study to investigate the association of TyG-BMI with bone mass and femoral neck geometry in healthy, nondiabetic, middle-aged and elderly individuals. The proximal femur, hip, and the lumbar spine were examined, as these are anatomical sites at high risk of osteoporotic fractures. As measures of bone strength, we used areal BMD (aBMD) and femoral geometry. TyG-BMI, which combines serum triglycerides, fasting plasma glucose, and obesity status, is considered more reliable than TyG for the identification of IR. It is a less expensive and more reliable marker of IR than traditional markers such as HOMA-IR [25,34]. Although IR cannot replace DXA in the diagnosis of osteoporosis, IR is introduced as a simple indicator, which can reflect the level and change in BMD. In addition, it can help to specify a reasonable lipid and FBG control target for nondiabetic individuals, because IR is known to have a deleterious effect on metabolic status, but this study found that it may have a protective effect on bone mineral density. In the future, we will further explore a reasonable range of lipids and FBG to achieve the greatest benefit.

We demonstrated that higher aBMD was associated with higher bone strength and lower fracture risk at all sites, with the association being significantly stronger in men than in postmenopausal women. The sex differences in these parameters may explain the higher incidence of fragility fractures in women. It also implies that sex-dependent femoral neck geometry contributes significantly to the ability to withstand stress.

The relationship between IR and BMD has been studied in different populations but the results have been mixed. Consistent with Riggs et al. [35], we found that higher TyG-BMI was associated with greater aBMD at both weight-bearing and nonweight-bearing skeletal sites, and TyG-BMI was significantly associated with osteoporosis. Previous studies have reported an association between bone metabolism and HOMA-IR, another surrogate marker of IR. Further, greater IR was found to be associated with higher BMD [36,37]. A study of Caucasian nondiabetic women from the Study of Women’s Health Across the Nation (SWAN) found that higher IR is associated with greater volumetric BMD and generally favorable bone microarchitecture at nonweight-bearing distal radius and weight-bearing distal tibia, independent of body weight [20]. This effect of hyperinsulinism on BMD may be because insulin exerts peripheral osteogenic effects via stimulation of osteoblasts or inhibition of osteoclasts. All these research results suggest that TyG-BMI is a protective factor for osteoporosis. However, Shin et al. [38] reported an inverse relationship between HOMA-IR and aBMD in a population-based study of young South Korean men (mean age, 49.9 years) suggesting that IR is a negative predictor of bone health. Our study population differs from that of Shin et al. [38] in several aspects: our study participants were Chinese, older (mean age, 60.3 years), and, importantly, nondiabetic. It is currently unknown whether the effects of IR or hyperinsulinemia on bone are age-, sex-, or race-specific. The inclusion of patients with diabetes in Shin et al. [38] may have confounded their results as HOMA-IR is unreliable in patients on antidiabetic medications; further, chronic hyperglycemia and/or antidiabetic medications may affect skeletal microarchitecture. Thus, comparison of their findings with ours is difficult.

In this study, we also investigated the association between TyG-BMI and femoral neck geometry. A recent study showed that several conditions associated with altered bone metabolism (for example, sarcopenia) result in poor femoral neck geometry, suggesting that these indices on DXA scans may be a good indicator of bone health [39]. Consistent with a previous Chinese study [40], we found that CSA, CT, SM, CSMI, and CSI decrease with age, whereas BR increases with age. These results imply that the decrease in CSA, CT, SM, CSMI, and CSI and increase in BR might contribute to fragility fractures of the femoral neck in old age. Further, CSA, CT, SM, CSMI, and CSI were lower, and BR higher, in women than in men, which may explain the greater vulnerability of the femoral neck in the former. 

There is a paucity of data describing the relationships between IR and femoral neck geometry. In the present study, TyG-BMI was positively associated with femoral neck CSA and SM, but negatively associated with BR; the relationships remained statistically significant even after controlling for age and previous fracture, suggesting that IR contributed to favorable femoral neck geometry. A possible explanation for this relationship is that insulin has an anabolic effect on bone, stimulating osteoblast growth and proliferation on periosteal surfaces and thus increasing SM and CSA. Overall, the results suggest that TyG-BMI has a positive effect on bone geometry in middle-aged and elderly Chinese individuals. Contrary to our findings, an inverse association between IR and bone size has been demonstrated in nondiabetic postmenopausal Caucasian women [20]. In Korean men and women, with and without diabetes, HOMA-IR and fasting insulin levels were found to be inversely associated with composite indices of femoral neck strength [41]. The primary difference between our study and the studies mentioned above is in the populations enrolled, implying that differences in sex, age, diabetes status, and race affect the relationship between IR and bone structure.

This study has some limitations. First, this was a retrospective study, so we cannot infer that IR leads to high bone strength; that will have to be clarified in longitudinal studies. Second, serum levels of hormones that affect bone metabolism (e.g., estrogen, androgen, and pituitary gonadotropin) were not evaluated in our study. Third, we did not use the HEGC technique and HOMA-IR for measuring IR, these methods, although accurate and popular, are time-consuming and costly and therefore not suitable for application in large samples. TyG-BMI is a reliable and highly sensitive alternative to HEGC [25] and HOMA-IR in nondiabetic individuals [33]. In addition, the results are only applicable to middle-aged and elderly nondiabetic Chinese individuals. Other populations need to be studied further.

In summary, TyG-BMI may be a reliable indicator of favorable bone density and strength in healthy, nondiabetic postmenopausal women and men and could be useful in the clinic to evaluate and predict the risk of osteoporosis. Possible mechanisms are as follows: physiological concentrations of insulin have been shown to inhibit osteoclast activity in bone and to increase osteoblast proliferation rate, collagen synthesis, alkaline phosphatase production, and glucose uptake. Insulin-deficient models are associated with reductions in mineralized surface area, osteoid surface, mineral apposition rate, and osteoblast activity and number [42]. Studies in murine models suggest that hyperinsulinemia and IR, in the absence of hyperglycemia, may contribute to reduced bone turnover, and reduced bone turnover may increase areal BMD [43]. IR and hyperinsulinemia can exist in both diabetic and nondiabetic people. In nondiabetic people who have IR and hyperinsulinemia, the risk of developing diabetes in the later stage is greatly increased. Additionally, IR and hyperinsulinemia are major cardiovascular risk factors in healthy persons and patients with diabetes [44]. However, considering the impact of IR on bone, it is particularly necessary to find an optimal cut point value. More mechanistic studies, both preclinical and clinical, are needed to better understand the effect of IR on bone and metabolism, and to clarify whether insulin resistance-related changes affect fracture risk.

## Figures and Tables

**Table 1:** Baseline characteristics of osteoporosis and nonosteoporosis group.

	Men		Women	
	Osteoporosis	Nonosteoporosis	Osteoporosis	Nonosteoporosis
Age (years)	62.0 ± 1.00 *	59.0 ± 0.00	65.0 ± 1.00 *	59.0 ± 0.00
Height (cm)	164.3 ± 0.90 *	167.7 ± 0.30	152.8 ± 0.80 *	156.5 ± 0.30
Weight (kg)	61.8 ± 1.20 *	70.7 ± 0.40	53.1 ± 0.80 *	57.9 ± 0.50
BMI (kg/m^2^)	22.9 ± 0.41 *	25.1 ± 0.13	22.7 ± 0.30 *	23.6 ± 0.18
Cholesterol (mmol/L)	4.87 ± 0.13	4.91 ± 0.05	5.08 ± 0.12 *	5.36 ± 0.06
Triglyceride (mmol/L)	1.57 ± 0.10	1.90 ± 0.07	1.37 ± 0.09	1.57 ± 0.06
HDL (mmol/L)	1.27 ± 0.04	1.23 ± 0.02	1.48 ± 0.04	1.50 ± 0.02
LDL (mmol/L)	3.10 ± 0.12	3.08 ± 0.04	3.20 ± 0.12	3.37 ± 0.05
FPG (mmol/L)	5.22 ± 0.14	5.33 ± 0.05	5.24 ± 0.09	5.30 ± 0.06
25(OH)D (nmol/L)	45 ± 2.00	46 ± 1.00	41.0 ± 2.00	43.0 ± 1.00
P (mmol/L)	0.86 ± 0.03	0.91 ± 0.01	1.04 ± 0.03	1.04 ± 0.02
Ca (mmol/L)	2.24 ± 0.03	2.25 ± 0.01	2.28 ± 0.03	2.26 ± 0.01
FN-BMD (g/cm^2^)	0.58 ± 0.01 *	0.76 ± 0.01	0.54 ± 0.01 *	0.69 ± 0.01
LS-BMD (g/cm^2^)	0.81 ± 0.02 *	0.98 ± 0.01	0.68 ± 0.01 *	0.88 ± 0.01
TH-BMD (g/cm^2^)	0.74 ± 0.01 *	0.93 ± 0.01	0.67 ± 0.01 *	0.83 ± 0.01
FN-CT (mm)	0.11 ± 0.00 *	0.15 ± 0.00	0.10 ± 0.00 *	0.13 ± 0.00
FN-SM (cm^3^)	1.32 ± 0.03 *	1.69 ± 0.02	0.94 ± 0.02 *	1.17 ± 0.01
FN-CSMI (cm^4^)	2.39 ± 0.07 *	3.05 ± 0.04	1.48 ± 0.04 *	1.86 ± 0.03
FN-CSI (g∙kg^−1^∙m^−1^)	3.43 ± 0.08 *	3.90 ± 0.03	3.23 ± 0.05 *	3.77 ± 0.04
FN-CSA (cm^2^)	1.99 ± 0.03 *	2.60 ± 0.02	1.62 ± 0.03 *	2.06 ± 0.01
FN-BR	16.6 ± 0.29 *	12.5 ± 0.08	15.7 ± 0.31 *	12.3 ± 0.12
MOF (%)	4.50 ± 0.30 *	2.50 ± 0.10	6.10 ± 0.30 *	3.60 ± 0.10
HF (%)	2.60 ± 0.30 *	0.60 ± 0.00	2.70 ± 0.20 *	0.70 ± 0.00
TyG index	8.69 ± 0.07	8.81 ± 0.03	8.54 ± 0.06	8.66 ± 0.03
TyG-BMI	199.1 ± 4.27 *	221.7 ± 1.56	193.8 ± 2.98 *	205.2 ± 1.89
Smoke, % (*n*)	46.7 (21)	34.8 (149)	1.1 (4)	0 (0)
Drink, % (*n*)	84.4 (38)	18.9 (66)	0.4 (1)	0 (0)
Previous fracture, (*n*)	8.9 (4)	6.5 (28)	15.3 (12)	13 (35)
Parental hip fracture, % (*n*)	6.7 (3) *	21.8 (54)	12.5 (10)	11.5 (32)

BMI, body mass index; FN: femoral neck; TH: total hip; LS: lumbar spine; BMD: bone mineral density; CT: cortical thickness; CSMI: cross-sectional moment of inertia; CSI: compression strength index; CSA: cross-sectional area; SM: section modulus; BR: buckling ratio; CT: cortical thickness; MOF: major osteoporosis fracture; HF: hip fracture; HDL high-density lipoprotein cholesterol, LDL: low-density lipoprotein cholesterol, FPG: fasting plasma glucose; TyG: triglyceride glucose index; TyG-BMI: combined TyG and BMI. * *p* < 0.05.

**Table 2:** Correlations of triglyceride glucose–body mass index and bone strength and risk of fracture.

	Men	Women
	r	r
FN-CSMI (cm^4^)	0.144 **	0.129 *
FN-CSI (g∙kg^−1^∙m^−1^)	−0.418 **	−0.408 **
FN-CSA (cm^2^)	0.219 **	0.190 **
FN-BR	−0.206 **	−0.119 *
FN-CT (mm)	0.237 **	0.185 **
FN-SM (cm^3^)	0.177 **	0.158 **
MOF	−0.076	0.05
HF	−0.178 **	−0.064

FN: femoral neck; CT: cortical thickness; CSMI: cross-sectional moment of inertia; CSI: compression strength index; CSA: cross-sectional area; SM: section modulus; BR: buckling ratio; MOF: major osteoporotic fracture; HF: hip fracture. * *p* < 0.05; ** *p* < 0.001

**Table 3:** Linear regression analysis between the TyG-BMI and the densitometry parameters.

		Men		
	Unadjusted	Model 1	Model 2	Model 3
		β	β	β
FN-CSI (g∙kg^−1^∙m^−1^)	−0.401 **	−0.414 **	−0.426 **	−0.426 **
FN-CSMI (cm^4^)	0.213 **	0.191 **	0.182 **	0.182 **
FN-SM (cm^3^)	0.238 **	0.212 **	0.205 **	0.204 **
FN-CT (mm)	0.263 **	0.238 **	0.230 **	0.231 **
FN-BR	−0.227 **	−0.202 **	−0.194 **	−0.195 **
FN-CSA (cm^2^)	0.2693 **	0.238 **	0.229 **	0.229 **
FN-BMD (g/cm^2^)	0.265 **	0.234 **	0.223 **	0.224 **
LS-BMD (g/cm^2^)	0.173 **	0.176 **	0.185 **	0.185 **
TH-BMD (g/cm^2^)	0.293 **	0.275 **	0.27 **	0.271 **
MOF	−0.067	−0.031	−0.055	−0.108
HF	−0.125	−0.078 *	−0.116 *	−0.141 *

Model 1: adjusted age; Model 2: adjusted age, smoke, drink; Model 3: adjusted age, smoke, drink, previous fracture, parental hip fracture. FN: femoral neck; TH: total hip; LS: lumbar spine; BMD: bone mineral density; CSI: compression strength index; CSMI: cross-sectional moment of inertia; SM: section modulus; CT: cortical thickness; BR: buckling ratio; CSA: cross-sectional area; MOF: major osteoporotic fracture; HF: hip fracture; TyG: triglyceride glucose index; TyG-BMI: combined TyG and BMI. ** *p* < 0.001; * *p* < 0.05.

**Table 4:** Linear regression analysis between the TyG-BMI and the densitometry parameters.

		Women		
	Unadjusted	Model 1	Model 2	Model 3
		β	β	β
FN-CSI (g∙kg^−1^∙m^−1^)	−0.409 **	−0.367 **	−0.367 **	−0/368 **
FN-CSMI (cm^4^)	0.147 *	0.194 **	0.194 **	0.194 **
FN-SM (cm^3^)	0.180 **	0.228 **	0.228 **	0.227 **
FN-CT (mm)	0.213 **	0.342 **	0.341 **	0.342 **
FN-BR	−0.167 **	0.283 **	0.283 **	0.282 **
FN-CSA (cm^2^)	0.2203 **	0.283 **	0.283 **	0.282 **
FN-BMD (g/cm^2^)	0.214 **	0.279 **	0.279 **	0.279 **
LS-BMD (g/cm^2^)	0.142 **	0.192 **	0.193 **	0.192 **
TH-BMD (g/cm^2^)	0.292 **	0.411 **	0.41 **	0.415 **
MOF	−0.015	−0.148 *	−0.148 *	−0.219 *
HF	−0.089	−0.293 **	−0.293 **	−0.309 **

Model 1: adjusted age; Model 2: adjusted age, smoke, drink; Model 3: adjusted age, smoke, drink, previous fracture, parental hip fracture. FN: femoral neck; TH: total hip; LS: lumbar spine; BMD: bone mineral density; CSI: compression strength index; CSMI: cross-sectional moment of inertia; SM: section modulus; CT: cortical thickness; BR: buckling ratio; CSA: cross-sectional area; MOF: major osteoporotic fracture; HF: hip fracture; TyG: triglyceride glucose index; TyG-BMI: combined TyG and BMI. ** *p* < 0.001; * *p* < 0.05.

**Table 5:** Multivariable logistic regression analyses between possible predictors and osteoporosis. Adjusted with age, sex, 25(OH)D, current smoker, current drinker.

	Unadjusted OR (95% CI)	*p*-Value	Adjusted OR (95% CI)	*p*-Value
Age	0.929(0.907, 0.950)	<0.001	0.919(0.892, 0.947)	<0.001
Sex, female	0.393(0.263, 0.587)	<0.001	0.486(0.266, 0.889)	0.019
History of parental hip fracture	0.823(0.434, 1.559)	0.550	0.872(0.414, 1.836)	0.719
Current smoker	0.784 (0.473, 1.730)	0.346	0.353(0.176, 0.709)	0.003
Current drinker	0.389 (0.176, 0.860)	0.020	1.588(0.608, 4.145)	0.345
25(OH)D	1.011(0.996, 1.027)	0.142	1.018(1.002, 1.034)	0.026
TyG-BMI	1.022(1.014, 1.029)	<0.001	1.019(1.010, 1.028)	<0.001

## Data Availability

The datasets used and/or analyzed during the current study are available from the corresponding author on reasonable request.

## Supplementary Materials

The following supporting information can be downloaded at: https://www.mdpi.com/article/10.3390/jcm11195694/s1, Table S1: Baseline characteristics of participants according to gender.

## Abbreviations

AGE	glycation end product
BMD	bone mineral density
BMI	body mass index
BR	buckling ratio
CSA	cross-sectional area
CSI	compression strength index
CSMI	cross-sectional moment of inertia
CT	cortical thickness
DXA	dual-energy X-ray absorptiometry
HEGC technique	hyperinsulinemic euglycemic clamp technique
HFs	hip fractures
HOMA-IR	homeostatic model assessment for insulin resistance
IR	insulin resistance
MOFs	major osteoporotic fractures
SM	section modulus
T2DM	type 2 diabetes mellitus
TyG-BMI	triglyceride and glucose–body mass index
