# Supplementary material for: Triglyceride Glucose–Body Mass Index Is a Reliable Indicator of Bone Mineral Density and Risk of Osteoporotic Fracture in Middle-Aged and Elderly Nondiabetic Chinese Individuals
Source: J Clin Med. 2022 Sep 26;11(19):5694. doi: 10.3390/jcm11195694 (PMC9572437; doi:10.3390/jcm11195694)
Supplement: Supplementary file 1 [file jcm-11-05694-s001.zip › jcm-1785477-supplementary.pdf]

Table S1. Baseline characteristics of participants according to gender.

	Men	Women	*p*-Value
Age (years)	59.0 ± 7.95	59.6 ± 7.72	0.126
Height (cm)	167.4 ± 5.68	155.8 ± 5.45	**<0.001**
Weight (kg)	69.9 ± 9.48	56.9 ± 7.69	**<0.001**
BMI (kg/m^2)	24.9 ± 2.82	23.5 ± 2.80	**<0.001**
Cholesterol (mmol/L)	4.90 ± 0.94	5.28 ± 0.98	**<0.001**
Triglyceride (mmol/l)	1.87 ± 1.37	1.51 ± 0.92	**<0.001**
HDL (mmol/l)	1.23 ± 0.30	1.49 ± 0.33	**<0.001**
LDL (mmol/l)	3.08 ± 0.85	3.33 ± 0.90	**<0.001**
FBG (mmol/l)	5.32 ± 0.95	5.27 ± 0.88	**0.449**
25 (OH)D (nmol/l)	46.2 ± 16.5	43.0 ± 15.0	**0.016**
P (mmol/L)	0.91 ± 0.11	1.04 ± 0.15	**<0.001**
Ca (mmol/l)	2.24 ± 0.09	2.26 ± 0.11	**0.065**
FN-BMD (g/cm^2)	0.75 ± 0.11	0.66 ± 0.1	**<0.001**
LS-BMD (g/cm^2)	0.96 ± 0.14	0.85 ± 0.14	**<0.001**
TH-BMD (g/cm^2)	0.91 ± 0.12	0.80 ± 0.11	**<0.001**
FN-CT (mm)	0.14 ± 0.02	0.13 ± 0.02	**<0.001**
FN-SM (cm^3)	1.65 ± 0.33	1.12 ± 0.22	**<0.001**
FN-CSMI (cm^4)	2.99 ± 0.75	1.78 ± 0.46	**<0.001**
FN-CSI (g·kg^{-1}·m^{-1})	3.85 ± 0.59	3.67 ± 0.59	**<0.001**
FN-CSA (cm^2)	2.55 ± 0.42	1.99 ± 0.32	**<0.001**
FN-BR	12.9 ± 2.11	12.9 ± 2.49	**0.812**
MOF (%)	2.71 ± 1.35	3.96 ± 1.99	**<0.001**
HF (%)	0.81 ± 1.04	1.09 ± 1.34	**0.003**
TyG index	8.80 ± 0.59	8.62 ± 0.53	**<0.001**
TyG-BMI index	219.6 ± 32.5	202.5 ± 29.8	**<0.001**
Smoke,% (n)	31.5 (170)	1.13 (4)	**<0.001**
Drink,% (n)	33.9 (104)	0.03 (1)	**<0.001**
Osteoporosis,% (n)	9.5 (45)	20.1 (72)	**<0.001**
Previous fracture,% (n)	7.2 (32)	15.1 (47)	**0.002**
Parental hip fracture,% (n)	13.7 (57)	13.3 (42)	**0.983**

BMI, body mass index; FN: femoral neck; TH:total hip; LS:lumber spine;BMD: body mineral density; CT: cortical thickness; CSMI: Cross sectional moment of inertia; CSI: compression strength index; CSA: Cross sectional area; SM: Section modulus ;BR: buckling ratio ; CT: Cortical thickness ; MOF: major osteoporosis fracture; HF: hip fracture; HDL-C high density lipoprotein cholesterol, LDL-C low density lipoprotein cholesterol, FPG fasting plasma glucose; TyG triglyceride glucose index, TyG-BMI combined TyG and BMI.
